# Assessment of lifestyle changes related to quarantine among Saudi population during the early COVID-19 pandemic: pre- and post-analysis

**DOI:** 10.1186/s42506-023-00149-1

**Published:** 2024-02-01

**Authors:** Mira M. Abu-Elenin, Ahmed A. Elshora, Marwa A. Shahin, Nesrin K. Abd El-Fatah

**Affiliations:** 1 Department of Public Health and Community Medicine, Faculty of Medicine, Tanta University, Tanta, Egypt; 2 Batterjee Medical College, Health Management Program, Jeddah, 21442 Saudi Arabia; 3 Department of General Surgery, Faculty of Medicine, Tanta University, Tanta, Egypt; 4 Batterjee Medical College, Medicine Practice Program, Surgery Department, Jeddah, 21442 Saudi Arabia; 5 Department of Maternal and Neonatal Health Nursing, Faculty of Nursing, Menoufia University, Shibin El Kom, Egypt; 6 Batterjee Medical College, Nursing Program, Jeddah, 21442 Saudi Arabia; 7 Department of Nutrition, High Institute of Public Health, Alexandria University, Alexandria, Egypt; 8 Taif Saudi Board of Preventive Medicine, Ministry of Health, Taif, Saudi Arabia

**Keywords:** COVID-19, Dietary pattern, Lifestyle. Pandemic, Physical activity, Quarantine, Saudi Arabia

## Abstract

**Background:**

The escalating emergence of the COVID-19 pandemic interrupted human life due to the ever-increasing morbidity, containment measures, and the associated emotional distress. This study examined the influence of COVID-19-related measures on the lifestyle behaviors of adults living in the Kingdom of Saudi Arabia (KSA).

**Methods:**

The study recruited 638 participants through convenience sampling in Jeddah and Taif cities, Saudi Arabia. Demographic characteristics and COVID-19-related information were collected through an anonymous self-reported electronic questionnaire. Lifestyle behaviors were assessed before and during the quarantine using the Healthy Dietary Habits Index (HDHI)-A and the International Physical Activity Questionnaire (IPAQ-SF).

**Results:**

The study revealed significant changes in the dietary pattern: a reduction in fish intake and increased consumption of French fries and candies. Vegetables and fruit intake increased significantly. Sedentary time > 6 h per day increased from 36.7% pre to 60.8% during the pandemic. Healthy dietary index score and physical activity MET-minutes/week values were respectively 3.5% and 37.9% significantly higher before compared to the full quarantine period.

**Conclusion:**

The pandemic detrimentally influenced eating habits and physical activity levels that led to weight gain, and hence higher vulnerability to COVID-19 infection and negative outcomes. This analysis provides public health agencies with data to tailor nutrition interventions that mitigate the observed adverse lifestyle behaviors.

## Introduction

The WHO Emergency Committee affirmed a global health emergency due to the COVID-19 pandemic on the 30th of January 2020 [1]. To tackle the spread of the COVID-19 pandemic, public health recommendations and governmental measures have stringently enforced lockdowns and restrictions. These restrictions interfered with individuals' routines practices, physical activity (PA), travel, and participation in various exercises [2]. To halt the rapid spread of COVID-19 in the Kingdom of Saudi Arabia, the government decided in the middle of March 2020 to enforce strict confinement measures, including banning mass gatherings, curfew, and partial and full quarantines stringently implemented across the entire country [3].

This unprecedented condition interrupted healthy eating and physical activity; for instance, limited access to daily grocery shopping which contributed to the decrease in the intake of fresh fruit, vegetables, and fish, in favor of highly processed ones like fast foods, unhealthy snacks, and ready-to-eat products, which are rich in sugars, salts and fats [4]. Furthermore, the psychological and emotional responses to the pandemic may induce disorderly eating attitudes and behaviors, commonly referred to as "Emotional eating" which is defined as overeating when experiencing trauma or negative emotions [5].

Additionally, the continuous follow-up of COVID-19 progression from various sources of media could be stressful, which in turn leads to overeating, especially comfort foods rich in sugar, which is defined as “food craving.” The simple carbohydrate-rich foods reduce stress since they stimulate serotonin secretion which has a positive influence on the mood [5]. Lengthy restrictions including e-Learning, remote work, banned social gatherings and partial closure of food suppliers disrupted daily activities and evoked boredom and anxiety, which might result in an increase in energy intake [6].

The change in food consumption seems to vary significantly across countries, partly due to the different restriction measures enacted by the different governments to confront the COVID-19 pandemic [7]. Moreover, in order to confront the negative experience of self-isolation, people looked for self-reward and physiological gratification through increased food consumption while ignoring signals of satiety and hunger [8]. Besides, staying at home for an extended time arouses feelings of monotony, which are often related to overeating as a method to overcome boredom [8, 9]. Conversely, this negative experience could lead to a restriction in food intake as a result of physiological stress reactions that mimic the internal sensation of satiety induced by eating [10].

Regarding the potentially compromised physical unfitness, it imposed a burden on population health which is proportional to the ability to cope with infections and the immunologic response [11]. Universally, physical inactivity and poor mental health are among the most important risk factors for major disease morbidity. This is not only true for the general population, but also for the elderly and patients with chronic diseases, as well as people who are at increased risk of COVID-19-induced mortality [12]. According to published data from a multinational survey, the COVID-19-related quarantine reduced all levels of physical intensity levels (overall, walking, moderate, and vigorous,). In addition, the daily sedentary time increased from 5 to 8 h per day [13]. Unhealthy dietary patterns such as overeating, frequent snacking, and skipping some main meals also increased with only alcohol binge drinking experiencing a significant decrease [13]. Similarly, other reports from Saudi Arabia and China indicated a decrease in the dietary quality index, increased rates of overweight/obesity, declined frequency of moderate/vigorous-intensity physical exercise, and increased sedentary, sleeping, and screen time due to COVID-19-related home confinement [14, 15]. On the other hand, a study in Italy detected a slight increase in levels of physical activity and a significant proportion of respondents tended to purchase organic or farmer fruits and vegetables, with increased adherence to the Mediterranean diet. However, most of the study subjects didn't change their eating habits (46.1%), while (16.7%) improved them and (37.2%) made them worse. Also, the smoking rate decreased during home isolation [16].

Throughout human history, the promotion of a healthy lifestyle has been challenging among the general population, and explanatory dietary data are required to mitigate the development of misconceptions regarding the association between certain diets and COVID-19 infection susceptibility and/or resistance [14].

Due to the lack of specific preventive measures against COVID-19 infection and certain pharmaceutical treatments for this virulent infection, people engaged in various preventive behaviors including the development of healthier dietary habits, eating nutritious food, and the elective uptake of micronutrient supplements known for their anti-inflammatory and protective properties (e.g., vitamins, minerals, probiotics, and nutraceuticals) particularly among at-risk groups such as the elderly and patients with chronic diseases [17].

The study question was “Did the COVID-19 related quarantine have an effect on some lifestyle behaviors of adults?” To the authors’ limited knowledge, evidence is scarce when it comes to the investigation of how human eating behavior and physical activity were affected by the constraints and pandemic-related lockdown in Saudi Arabia. Therefore, this study aimed to assess the changes in lifestyle behaviors induced by the COVID-19-related quarantine and lengthy restrictions among the Saudi population who live in Jeddah and Taif cities, in the Kingdom of Saudi Arabia.

## Methods

### Study design and setting

A retrospective cross-sectional study was conducted in the Kingdom of Saudi Arabia in Jeddah and Taif cities, during the early COVID-19 pandemic from 1st February to the end of May 2020.

### Study participants and sampling

A convenience sampling technique was used to recruit Saudis aged 18 years and over who live in Saudi Arabia during the period of the study. Exclusion criteria: we excluded residents of other nationalities, Saudis who reside outside the country, pregnant women, and those who expressed their unwillingness to participate in the study. Owing to the pandemic situation, they were kindly asked to participate via online announcements on popular social media platforms (such as Twitter, Instagram, WhatsApp, and Facebook) as well as through the researchers’ groups network with a request to circulate the survey broadly with their contacts to recruit more participants. A brief description of the study’s aim and the declaration of confidentiality and anonymity were given to the participants before enrollment in the study.

The sample size was computed using the EPI-INFO software, and a minimum required sample of 384 participants was determined, assuming that the prevalence of healthy dietary habits change during the pandemic is 50%, with a precision of 5% and a confidence level of 95%.

A total of 730 questionnaires were completed and only 638 (87.4%) were valid. Respondents who were less than 18 years of age, not living in Saudi Arabia, or did not complete the questionnaire appropriately were excluded from the final analysis.

### Study tools

Participants voluntarily reported their sociodemographic data and completed validated questionnaires about eating habits (Healthy Dietary Habits Index for adults (HDHI)-A) and physical activity (International Physical Activity Questionnaire: Short Form [IPAQ-SF]) [18, 19]. Bodyweight, weight perception, routine diet regimen, and history of smoking cigarettes or other tobacco products were also addressed. The previous data was collected regarding the situation before the imposed quarantine (a month before COVID-19 was first spotted in Saudi Arabia on March 2nd, 2020), and during the quarantine (before the lockdown was lifted on June 1st, 2020). Financial distress, previous infection, direct exposure to COVID-19 infection, and COVID-related sources of information were also assessed.

#### Healthy Dietary Habits Index for adults (HDHI)-A

Dietary intake habits data was collected using a Diet Quality Index or Healthy Dietary Habits Index questionnaire. It is a valid tool for ranking diet quality among adults. It is composed of 15 items with a score ranging from 0 (least healthy) to 4 (most healthy response) for each item. The dietary pattern of respondents was examined considering: their routine meal plans, preferred methods of cooking, food frequency intake of each food group, and frequency of consumption of various beverages. Items from 1 to 7 are assigned to food preparation, choices, or habits related to fat intake from different foods. Items 8–10 concern dietary fiber, bread, fruits, and vegetable food groups. Participants reported their usual intake of a standard serving size which was demonstrated in the text of questions (e.g., an ounce of animal protein, 1 cup of raw or cooked vegetables or vegetable juice, 2 cups of raw leafy salad greens, 1 cup of fruit, ½ cup of dried fruit, 1 cup of 100% fruit juice, one ounce of bread or rice) of fruit, vegetables, and grains per day. They were also asked to report the number of servings of legumes and nuts they consumed per week (½ ounce of nuts and ¼ cup of cooked legumes were equal to one serving). Item 11 captures the intake of added sugar and sugar-sweetened beverages. The frequency of breakfast and fast-food consumption was assessed in items 12 and 13, respectively. Item 14 was related to adding salt to foods before eating and it was reversely scored. While item 15 asked about the use of low-salt products. The total HDHI-A was a summation of scores from the 15 items and ranged from 0 to 60. A greater total score reflects a healthier dietary pattern. The same habits were assessed before and during the pandemic. The tool is written originally in English and the principal investigator translated it into Arabic, then the translated Arabic version was back-translated into English to ensure the language integrity.

#### International Physical Activity Questionnaire: Short Form [IPAQ-SF]

Physical activity was assessed before and during the pandemic. The IPAQ-SF is valid in several countries. It consists of 7 questions and collects information on the time (i.e., number of days and average time per day) spent being physically active and measures vigorous-intensity activity, moderate-intensity activity, walking activity, and sitting on a typical day. The IPAQ-SF results were reported as low-, moderate- or high-PA levels and continuous total metabolic equivalents (METs) minutes per week according to its scoring protocol. Total weekly physical activity (MET-Min week) was calculated by multiplying the number of minutes spent in each activity category by the specific MET score or MET intensity values for each activity. While sedentary times were subjectively reported in terms of minutes per day. PA levels were summarized as; low, moderate, and high.

#### Anthropometry

Height and weight data were obtained by self-reporting of the study participants. Body mass index (BMI) was then calculated as the ratio of weight in kilograms to height in meters squared. BMI was classified according to the WHO classification as follows: underweight (BMI < 18.5), normal weight (BMI from 18.5 to 24.9), overweight (BMI between 25 and 29.9), and obese (BMI ≥ 30) [20].

### Statistical analysis

The data management process was conducted using SPSS for Microsoft Windows version 23. Explanatory variables including demographic characteristics and COVID-19-related data were presented as frequency and percentages in tables and figures. Descriptive statistics demonstrated the scores for the (HDHI)-A, (IPAQ-SF), and the anthropometric data. According to the results of the Kolmogorov-Simonov normality test, Paired *t*-test was deployed in the comparison analysis between outcome variables before and during the quarantine. While for comparing the nonparametric variables Wilcoxon rank test was used instead. The level of significance in this study was adopted at a* p*-value < 0.05.

## Results

### Sample description

The present study included 638 respondents; their mean age was 35.04 ± 7.24 years and ranged from 18 to 71 years. Most of the respondents were female (65.8%) and (34.8%) were married. More than one-third attained a high educational level (37.5%). The participants’ proportion with a governmental job was equal to the nongovernmental ones (31% each). The participants whose income was more than 15,000 SAR/month presented (46.1%), and (32.6%) mentioned that their financial status was negatively affected as a result of circumstances related to the COVID-19 quarantine. Regarding smoking status, about one-fifth were smokers (21%) which decreased during the quarantine to (20.2%). Half of the smokers reported smoking cigarettes and shisha mainly (Tables 1 and 2).

**Table 1:** Demographic characteristics of the study participants in Jeddah and Taif cities during the early COVID-19 pandemic (*n* = 638)

Characteristics	No.	%
**Gender**		
- Male	218	34.2
- Female	420	65.8
**Age** (years)		
- 18–25	98	15.3
26–35	137	21.6
- 36–45	159	24.8
46–55	124	19.5
56–65	82	12.8
66 +	38	6
**Marital status**		
- Single	222	34.8
- Married	364	57.1
- Widow/divorced	52	8.1
**Educational level**		
- Low	56	8.5
- University	344	54.0
- Higher	238	37.5
**Job**		
- Governmental employee	198	31
- Non-Governmental employee	198	31
- Business	6	0.9
- Student	98	15.4
- Retired	30	4.7
- Housewife/Unemployed	108	17.0
**Income (SAR/month)**		
- Less than 3000	30	4.7
- 3000–	72	11.3
- 6000–	108	16.9
- 10,000–	134	21
- More than 15,000	294	46.1
**Financial status affected due to COVID-19**		
- Not affected	348	54.5
- Decreased	208	32.6
- Improved	82	12.9
**Smoking history**		
- Non-smoker	504	79
- Smoker	134	21
**Type of smoking (*n* = 134)**		
- Cigarettes only	58	43.2
- Shisha only	56	41.8
- Others/mixed	20	15
**Frequency per day**		
- 20 times +	16	12
- 11–19 times	18	13..3
- 5–10 times	24	18.0
- Less than 5 times	76	56.7
**Medical history**		
- Negative	488	76.5
- Positive	150	23.5
**COVID-19 prior-diagnosis**		
- Negative	624	97.8
- Positive	14	2.2
**Contact with cases with confirmed COVID-19**		
- No	542	85
- Yes	96	15

**Table 2:** Body weight-related data of the study individuals in Jeddah and Taif pre and during the early COVID-19 pandemic (*n* = 638)

Variable	Pre COVID-19 quarantine	During COVID-19 quarantine	Test of significance	*p*-value
**Bodyweight** (mean ± SD, range)	72.7 ± 16.8 (35–145)	73.67 ± 18.9 (35–172)	*t* = 2.6^a^	0.007*
**BMI** (mean ± SD, range)	26.9 ± 5.5 (13.8–45.8)	27.2 ± 6.19 (14.2–62.5)	*t* = 2.5^a^	0.01*
**BMI**				
- Underweight	31 (4.9)	30 (4.7)	*Z* = − 2.3^b^	0.017*
- Normal weight	215 (33.7)	202 (31.7)		
- Overweight	212 (33.2)	216 (33.9)		
- Obese	180 (28.2)	190 (29.8)		
**Practice dieting**				
- Yes	178 (27.9)	156 (24.5)	*χ*^2^ = 2.5^c^	0.11
- No	460 (72.1)	482 (75.5)		

^a^ *t*-test, ^b^ Wilcoxon rank test, ^c^ McNemar test, * Statistical significance

**Fig. 1:**
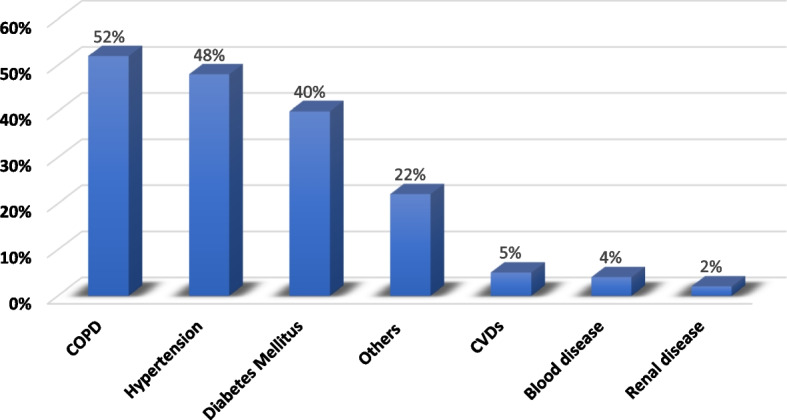
Distribution of medical morbidities among study participants with positive medical history in Jeddah and Taif cities during the early COVID-19 pandemic (*n* = 150)

The medical history indicates that 20.2% had a prior COVID-19 infection and 15% reported direct contact with confirmed cases. The medical history of participants is shown in Fig. 1.

**Fig. 2:**
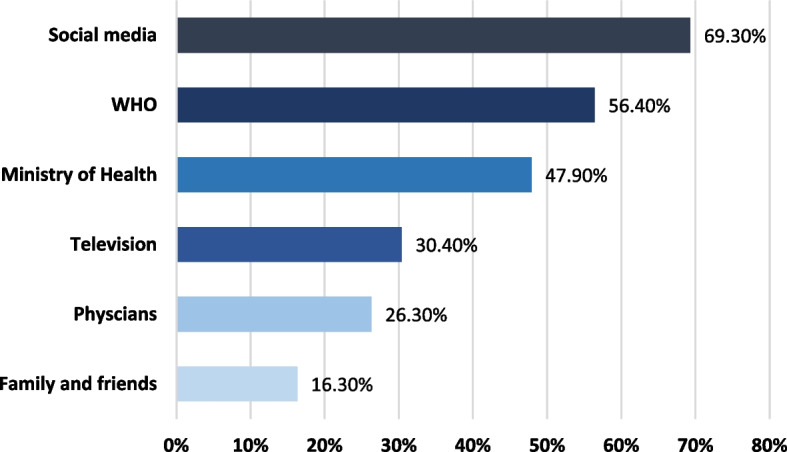
Sources of the COVID-19-related information as reported by the study individuals in Jeddah and Taif cities during the early COVID-19 pandemic (*n* = 638)

Figure 2 illustrates the reported sources of the COVID-19 pandemic-related information, the most frequently reported sources were social media (69.3%), followed by the WHO newsletter (56.4%).

### Perceived bodyweight concerns and BMI before and during the quarantine

**Fig. 3:**
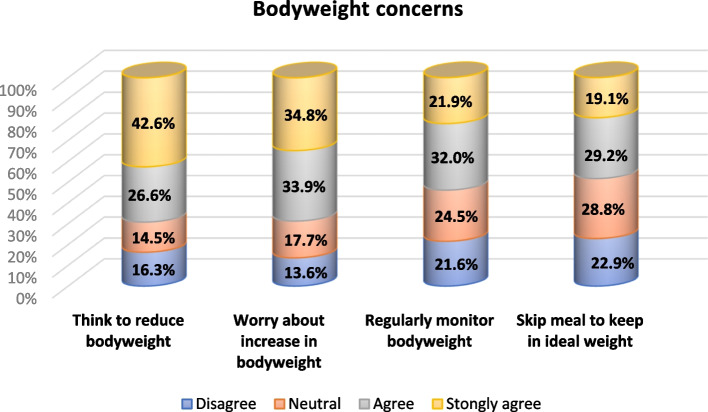
Bodyweight concerns of the study individuals in Jeddah and Taif cities during the early COVID-19 pandemic (*n* = 638)

The study revealed a statistically significant increase in the proportion of overweight and obesity among participants (pre 33.2% and 28.2%; post 33.9% and 29.8%). A considerable proportion of participants reported practicing a restrictive dietary regimen (72.1% pre; 75.5% post). However, more than one-third of participants strongly agreed that they should reduce their weight and worried about any increase in their body weight (42.6% and 34.8%, respectively) as shown in Fig. 3.

### Dietary habits before and during the quarantine

**Fig. 4:**
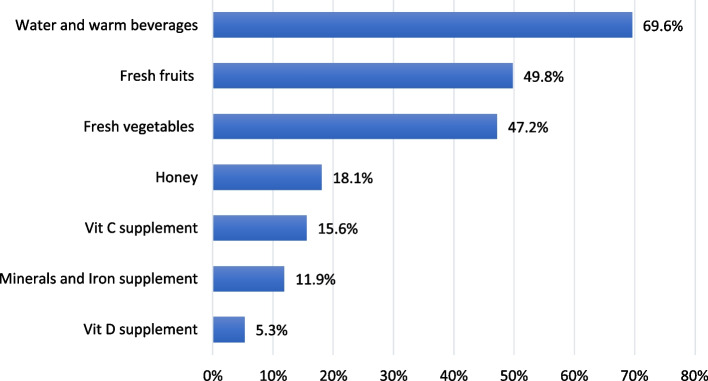
The commonly consumed food and dietary supplements by the study individuals in Jeddah and Taif cities during the early COVID-19 pandemic (*n* = 638)

The consumption of water/hot beverages, fresh fruits, and vegetables has significantly increased between 69.6%, 49.8%, and 47.2%, respectively, of the participants during the COVID-19 imposed quarantine (Fig. 4). The comparison of eating habits pre and during the quarantine indicated a significant increase in the frequency of consumption of the following items: fast food, French fries, and candies (*p* =  < 0.0001, 0.005, 0.02 respectively). On the other hand, there was a significant reduction in fresh fish consumption and intake of sugar-free drinks (*p* < 0.0001, = 0.008 respectively). However, a statistically significant improvement in fruit and vegetable intake was detected,17.6% and 3.6% of the participants used to consume 3–4 servings/day before the pandemic and they increased to 21.5% and 6.1% respectively during the quarantine (*p* = 0.0.4). Also, there was a significant improvement in some healthy habits including cooking methods, trimming the fat from meat and chicken, lower use of additional salt, and purchasing low-salted food products (*p* =  < 0.0001, 0.002, 0.0008, 0.04, 0.0001, respectively) (Table 3).

**Table 3:** Dietary habits of the study participants in Jeddah and Taif cities pre and during the early COVID-19 pandemic (*n* = 638)

Healthy dietary index	Pre COVID-19 quarantine		During COVID-19 quarantine		Test of significance (95%CI)	*p-value*
	**No.**	**%**	**No.**	**%**		
**Breakfast consumption: /week**						
Never	54	8.5	70	11.0	− 0.825^**a**^ (0.37–0.43)	0.4
1–2 times	110	17.2	90	14.1		
3–4 times	102	16.0	112	17.6		
5–6 times	164	25.7	164	25.7		
- Everyday	208	32.6	202	31.6		
**Fast food consumption/week**						
≥ 7 times	27	4.2	30	4.7	− 10.14^**a**^ (0.0001–0.003)	< 0.0001*
5–6 times	41	6.4	66	10.3		
3–4 times	40	6.3	132	20.7		
1–2 times	222	34.8	276	43.3		
< once	308	48.2	134	21.0		
**Animal protein consumption/week**						
≥ 7 servings	74	11.6	64	10.0	− 0.898^**a**^ (0.36–0.38)	0.37
5–6 servings	122	19.1	148	23.2		
3–4 servings	234	36.7	224	35.1		
1–2 servings	154	24.1	150	23.5		
< one serving	54	8.5	52	8.2		
**Meat fat trimming**						
- Never	58	9.1	50	7.8	− 3.14^**a**^	0.002*
- Rarely	44	6.9	38	6.0	(0.001–0.002)	
- Sometimes	148	23.2	146	22.9		
- Regularly	214	33.5	222	34.8		
- Always/do not eat meat	174	27.3	182	28.5		
**Chicken fat trimming**						
- Never	60	9.4	50	7.8	− 3.07^**a**^	0.002*
- Rarely	34	5.3	38	6.0	(0.001–0.002)	
- Sometimes	156	24.5	158	24.8		
- Regularly	212	33.2	206	32.2		
- Always/do not eat chicken	176	27.6	186	29.2		
**Eating processed meat/week**						
≥ 7 times	12	1.9	10	1.6	− 0.2^**a**^	0.9
5–6 times	8	1.3	10	1.6	(0.98–0.9)	
3–4 times	292	45.8	298	46.7		
1–2 times	114	17.9	106	16.6		
< once/never	212	33.1	214	33.5		
**Fish consumption/week**						
Never	110	17.2	232	36.4	− 9.4^**a**^	< 0.0001*
< once	234	36.7	202	31.7	(0–0.001)	
Once	208	32.6	130	20.3		
2 times	76	11.9	70	11.0		
3–4 times	2	0.3	2	0.3		
> 4 times	8	1.3	2	0.3		
**Cooking method**						
- Deep frying	192	30.1	168	26.3	− 18.93^**a**^	< 0.0001*
- Grilling	278	30.6	288	45.1	(0–0.001)	
- Boiling	128	20	136	21.3		
- Steaming	40	6.3	46	7.3		
**Choosing low-fat food**						
- Never	114	17.9	128	20.1	− 1.82^**a**^	0.06
- Rare	118	18.5	100	15.7	(0.061–0.073)	
- Sometimes	224	35.1	240	37.6		
- Regular	94	14.7	92	14.4		
- Always	88	13.8	78	12.2		
**Type of used cooking fat**						
- Margarine	22	3.4	22	3.4	− 1.35^**a**^	0.17
- Butter	96	15.2	112	17.6	(0.17–0.18)	
- Vegetable oil	312	48.8	298	46.7		
- No oil/olive/flaxseed oil	208	32.6	206	32.3		
**Milk consumption/day**						
No/ < one cup	282	44.2	286	44.8	− 0.63^**a**^	0.6
1cup/day	280	43.9	278	43.6	(0.59–0.61)	
≥ 2 cups/day	76	11.9	74	11.6		
**French fries/week**						
≥ 7 times	8	1.3	16	2.5	− 2.75^**a**^	0.005*
5–6 times	24	3.8	52	8.2	(0.004–0.007)	
3–4 times	160	25.1	136	21.3		
1–2 times	282	44.2	272	42.6		
Never/ < once	164	25.6	162	25.4		

^a^ *z* value of Wilcoxon rank test , * Statistical significance

### Physical activity before and during the quarantine

The frequency of moderate and high-intensity PA significantly decreased during the quarantine than before its emergence (*p* = 0.006,0.0001, 0.008, 0.0002, respectively). The proportion of participants who reported sitting for more than 6 h per day significantly increased from 36.7% pre to 60.8% during the quarantine (Table 4).

**Table 4:** Physical activity level of the study participants in Jeddah and Taif cities pre and during the early COVID-19 pandemic (*n* = 638)

Physical activity item	Pre COVID-19 quarantine		During COVID-19 quarantine		Test of significance (95%CI)	*p-*value
	**No.**	**%**	**No.**	**%**		
**Frequency of moderate-intensity PA days/week**						
Never	200	31.3	234	36.7	− 2.7^**a**^ (0.005–0.008)	0.006*
1 day	94	14.7	96	15.0		
2 days	60	9.4	94	14.7		
3 days	146	22.6	92	14.4		
4 days	40	6.2	40	6.2		
5 days	44	6.7	28	4.4		
6 days	20	3.1	26	4.1		
7 days	34	5.0	28	4.5		
**Duration of moderate-intensity PA**						
Never	248	38.9	176	27.6	− 6.21^**a**^ (0–00.1)	0.0001*
10–<20 min	94	14.7	112	17.6		
20–<30 min	144	22.6	114	17.8		
30–<60 min	118	18.5	168	26.3		
60 min +	34	5.3	68	10.7		
**Frequency of high-intensity PA d/wk**						
- Never	350	54.9	380	59.6	− 2.6^**a**^ (0.005–0.008)	0.008*
1 day	88	13.8	92	14.4		
2 days	60	9.4	58	9.1		
3 days	64	10.0	38	6		
4 days	20	3.1	34	5.3		
5 days	24	3.8	10	1.6		
6 days	20	3.1	12	1.9		
7 days	12	1.9	14	2.2		
**Duration of high-intensity PA**						
Not at all	343	53.8	398	62.4	− 3.6^**a**^ (0.0001–0.001)	0.0002*
10–<20 min	112	17.6	84	13.2		
20–<30 min	71	11.1	80	12.6		
30–<60 min	92	14.4	61	9.6		
60 min+	20	3.1	14	2.2		
**Sedentary/resting time/day**						
> 6 h	234	36.7	388	60.8	11.7^**a**^ (0–0.001)	< 0.0001*
5–6 h	180	28.2	140	21.9		
3–4 h	150	23.5	68	10.7		
1–2 h	74	11.6	42	6.6		

^a^*z* value of Wilcoxon rank test , * Statistical significance

### Lifestyle changes before and during the quarantine

As demonstrated in Table 5, the total score of the HDHI- A was 3.5% higher before, compared to during the pandemic. The study detected a significant decrease in the MET values of moderate-intensity PA during the quarantine, compared to prior to its implementation by 47.4%. However, vigorous-intensity PA MET values increased by 34.1% during the quarantine. The overall MET values for all PA levels decreased significantly by 37.9% during the quarantine time.

**Table 5:** Dietary habits index and physical activity scores of the study participants in Jeddah and Taif cities pre and during the early COVID-19 pandemic (*n* = 638)

Variable	Pre COVID-19 quarantine		During COVID-19 quarantine		Test of significance (95% CI)	Δ(Δ%)	*p*-value
	**Median (IQR)**	**Mean ± SD** **range**	**Median (IQR)**	**Mean ± SD** **Range**			
**Healthy dietary index**	31 (27–35)	30.49 ± 6.32 (6–47)	30 (26–34)	29.41 ± 6.1 (6–44)	*t* = 6.68^a^ (0.76–1.39)	1.08(3.5)	< 0.0001*
**Total PA MET/week**	110 (0–748)	636.87 ± 1196.2	0(0–329)	395.47 ± 1020.8	*Z* = − 6.69^b^ (0–0.01)	241.4(37.9)	< 0.0001*
**PA intensity MET/week**					*z* =		
- Walking	0 (0–247)	181.1 ± 343.7	0 (0–99)	99.6 ± 233.4	− 5.79^b^	81.5 (44.9)	0.0001*
- Moderate intensity	0 (0–300)	230.4 ± 437	0 (0–120)	121.2 ± 283	− 6.1^b^	109.2 (47.4)	0.0001*
- Vigorous	0 (0–130)	225.26 ± 606	0 (0–200)	302.2 ± 740.4	− 3.9^b^	76.94 (34.1)	0.008*
**Physical activity level**	***n***	***%***	***n***	***%***			
- Low	470	73.7	506	79.3	*z* = 3.64^b^	––-	0.0002*
- Moderate	90	14.1	86	13.5	(0–0.001)		
- High	78	12.2	46	7.2			

Δ mean change, ^a^paired *t*-test, ^b^Wilcoxon rank test, * Statistical significance

## Discussion

The COVID-19 pandemic had a profound impact on public health and resulted in severe economic and social distress at the global level [1]. Recent research has documented deterioration in dietary habits [21] and lifestyle behaviors during the pandemic [11, 13]. The present study compared the patterns of food and beverage consumption as well as physical activity and smoking before and during the quarantine among 638 Saudis.

We detected a detrimental effect of the COVID-19-related quarantine on dietary habits and practice of physical activity among the responders. A significant change in food and beverage consumption in response to the quarantine was observed, including more frequent consumption of fast food, French fries, and candies and less frequent consumption of fish and sugar-free drinks. That was expected since there was limited access to groceries, food insufficiency in stores, and an interrupted food supply chain during the early waves of the pandemic.

Evidence has documented that junk and processed food provoke inflammation and oxidative stress which increases vulnerability to diseases. While fresh foods are rich in antioxidants which have protective effects [21].

The current study observed a remarkable change in the frequency of consuming fast food; where 6.3 and 6.4% reported having junk food 3–4 and 5–6 times per week before the quarantine; then increased to 20.7% and 10.3% respectively during the quarantine. Though, this happened in spite of the fact that maintaining a healthy diet was highly recommended during this serious pandemic period to support the immune system [22, 23]. To our knowledge, during the precautionary measures taken by the Kingdom, restaurants were not completely closed at any stage. Restaurants were closed only during the curfew time which lasted from March 24th till it was opened completely on May 31st. The observed increase of fast food consumption does not align with the findings of a study conducted in Kuwait [24] which found a significant reduction in the frequency of fast-food consumption as a result of fears regarding the transmission of COVID-19, whether it is from unhygienic practices at restaurants, or the delivery driver or people’s desire to eat healthier during the pandemic. Factors such as unexpected lifestyle changes, lockdown, isolation, anxiety, fear, stress, and depression can predispose to unhealthier food choices [25, 26].

Consistent with our findings, previous studies conducted in Saudi Arabia verified the negative impact of the lockdown on healthy dietary patterns and physical activity [25, 26]. This was not consistent with the findings of Di Renzo et al. (2020) who found a better adherence to healthy types of cooking during the COVID-19 quarantine among the Italian population [27].

Though it was insignificant, we observed an increase in the rate of skipping breakfast among participants during the quarantine. This may be understandable, considering that during the study period, there was a partial lockdown and lengthy restrictions at home, which might have led to staying up late at night and oversleeping during the day. This was consistent with Okada et al. 2021 who found a significant association between late dinner or bedtime snack and skipping breakfast [28]. Increased consumption of candies and sugar was evident in the current study, where the (15%) of participants who used to eat candies 5–6 times/day increased to (19.4%). A similar finding was reported by Scarmozzino and Visioli [29], who found that half of the participants of an Italian sample reported an increase in the consumption of sweet foods during the COVID-19 quarantine. This may be due to the stress and anxiety induced by the enforced quarantine and pandemic waves which might trigger a higher consumption of sugary foods [5, 30, 31].

Regarding other food groups, there were no significant changes in terms of red meat, chicken, type of fat, milk, grains, legumes, and nuts consumption, before and during the quarantine. Meanwhile, the present study revealed a significant improvement in some healthy dietary habits including cooking methods, trimming the fat from meat and chicken, lower use of additional salt, purchasing low-salted food, and a considerable increase in fruit and vegetable intake. However, the total healthy eating index score, was 3.5% significantly higher before, compared to during the quarantine period, indicating its adverse effect on dietary quality.

Obesity and deficiency of Vit E, Vit C, Beta carotene, and antioxidants are potentially associated with impaired immunological responses and more susceptibility to the contagion of infectious diseases [22].

It was expected to find a reduction in the consumption of fresh vital food, accompanied by micronutrient deficiency. Interestingly, the consumption of fresh fruits, vegetables, and water/hot beverages among the study participants significantly increased during the quarantine despite of enormous difficulties in the supply of agricultural products early and amid the pandemic [7].

This favorable behavior might be attributed to the prevalent awareness about being sufficiently hydrated and the benefits of eating fruits and vegetables as sources of vitamins and minerals to combat the risk of infection [32].

Likely, findings of a similar study, reported that the majority of the study population had increased their water intake and more than one-third (37.4%) reported eating healthier food including fruits and vegetables [27]. Comparable to our results, we detected a much higher sweets and junk food intake, which are rich in simple sugar and saturated fatty acids [30]. This explains the significant decline of the healthy dietary index observed during the quarantine.

As for the consumption of fish, it was significantly lower than before the quarantine. Similar findings were also reported among Chinese and Kuwait residents by Zhao et al. (2020) [25] and Husain W et al. (2020) [24]. A possible explanation for this behavior includes fish unavailability due to the closure of the fish markets early in March 2020 according to the precautionary measures taken by the Kingdom Public Health Authority (WAQYA).

Unsurprisingly, the most commonly consumed dietary supplements were: vitamin C, iron with minerals, and vitamin D supplements (15.6%, 12%, 5.3%) as shown in Fig. 4, since they are well-known for their immune-boosting effects [33]. A similar pattern of dietary supplement intake was observed by Bakhsh et al. (2021) in KSA [34].

The current study reported a weight gain of around 1 kg (mean BMI; before 72.7 ± 16.8 to during 73.67 ± 18.9 kg/m^2^). Similarly, Bakhsh et al. (2021) detected a higher weight gain average of ~ 3–5 kg during the quarantine time [34]. This was also consistent with other studies conducted in Italy and Poland [24, 29, 35].

Obesity causes low-grade inflammatory status, elevates adipokine levels released from adipose tissue, and modulates immunological responses [36]. That may induce metabolic disorders such as insulin-dependent diabetes mellitus (IDDM), dyslipidemia, and hypertension. These immunomodulatory effects in the natural and acquired immunity make the human body more prone to infections, alter the response to antiviral and antimicrobial medication, and reduce the immune response to vaccination [37]. The resulting immunological changes could predispose to an exaggeration of the respiratory COVID viral infections [38].

Consistent with the literature, unhealthy meal patterns, and sedentary lifestyle behaviors are likely associated with weight gain [36, 39]. Moreover, the emotional distress accompanied by being locked at home for months and fears of the novelty and the vast spread of COVID-19 might provoke emotional eating and food cravings [7].

The current study found that 42.6% strongly agreed to reduce their body weight and 34.8% strongly worried about any increase in their weight. A much lower proportion was reported from a population survey in Italy, which indicated that 14% believed that they should lose weight and 34.4% reported elevated hunger sensations during the lockdown [27]. This discrepancy could be attributed to sociocultural norms diversity.

Though the weight gain-related concerns among responders were high as shown in Fig. 3; our analysis detected a significant increase in body weight during the quarantine. Emotional eating, negative changes in eating habits, and declined PA levels could rationalize the observed weight gain. This obesogenic environment might contribute to vulnerability to COVID-19 infection and worsen the prognosis [38].

Considerably, more than two-thirds (69.3%) of the study individuals received COVID-19-related guidelines for prevention and control through social media platforms, which necessities-fostering the role of reliable health resources such as the WHO, health authorities, and scientific periodicals to enhance the population health awareness.

Of note, about 8% (*n* = 11) of smokers reported that they reduced the frequency of smoking below the amount they used to, and only 3% (*n* = 4) of them quit smoking during the quarantine period. Consistently, an Italian population survey in 2020 reported that 3.3% of smokers stopped smoking during the early pandemic and their smoking rates decreased by 0.5% [27].

This phenomenon might be explained by the fact that during this period people started working from home and would not expose their households to secondhand smoke. Also, the fear associated with smoking hazards may be a factor, as it increases the susceptibility to respiratory complications and the mortality risks of COVID-19 [2]. This is because tobacco smoking stimulates the angiotensin-converting enzyme type 2 receptors which are potential adhesion localities for novel coronavirus SARS-CoV-2 [40].

Regarding the physical activity level, our results indicated that nearly two-thirds of the study participants were physically inactive; consistent figures were reported by the Saudi national survey among the general population in 2020 [41]*.* However, the low levels of PA were exacerbated by the restrictive measures related to the pandemic, where there was a significant reduction of mean total PA MET/week compared to the pre-quarantine times. Similar results were detected in other related studies [13, 42].  Our findings are supported by recent evidence from local and international observations showing a universal decline in all physical activity levels during the COVID-19 lengthy measures [13, 26, 43]. Since the quarantine restricted the people’s mobility to go to work, gym, parks, and even practice normal daily chores, the observed reduction of PA was anticipated.

### Strengths and limitations

Several limitations are noticed in the present study. First, the use of a self-reported questionnaire which was used due to the specific circumstances of the pandemic, since remote data collection using social networks was more feasible and necessary. Second, all participants were asked to report their daily lifestyle habits before the imposed quarantine, which may be subjected to recall bias. Third, convenience sampling may have led to selection bias, thus a probability sampling technique is required to ensure the generalizability of findings by minimizing the potential for bias. Lastly, this study was also limited by its cross-sectional design, which precludes the investigation of causal relationships.

Despite those limitations, there were some important strength points. To the best of our knowledge, this is the first study that documented the preliminary dietary changes in Saudi Arabia, during the implemented quarantine due to the COVID-19 pandemic. This study has public health implications as it can provide background information to public health agencies. The usage of validated questionnaires for assessing the healthy eating index for adults and physical activity was one of the strong points as well. The design of the online questionnaire used, where the questions about lifestyle practices before and during the COVID-19 lockdown were placed next to each other for better recalling and comparing was also a strength point.

## Conclusion

The preliminary results of the study indicated that the COVID-19-related quarantine adversely impacted lifestyle behaviors, with a significant reduction in PA level, increased consumption of French fries and fast food, and reduction of weekly fish consumption. However, there was a significant improvement in fruit and vegetable intake. Collectively COVID-19-related lockdown has a negative impact on healthy eating index, indicating its adverse effect on dietary quality.

It is recommended to monitor the unhealthy lifestyle consequences to be integrated within the consistent surveillance system during pandemics. Additionally, policymakers should consider nutrition education interventions and reducing the obesogenic environment within the context of public health response to such a crisis, in order to re-adjust, restore, and maintain healthy eating and potent living practices.

## Data Availability

The data set for the current study are available upon a reasonable request to the corresponding author.

## Abbreviations

BMI: Body mass index
COVID-19: Coronavirus disease
COPD: Chronic obstructive pulmonary disease
CVDs: Cardiovascular diseases
HDHI-A: Healthy Dietary Habits Index for adults
IDDM: Insulin-dependent diabetes mellitus
IPAQ-SF: International Physical Activity Questionnaire
KSA: Kingdom of Saudi Arabia
METs: Metabolic equivalents
PA: Physical activity
WHO: World Health Organization
